# Diagnostic accuracy of 1,000 endorectal ultrasounds before transanal endoscopic microsurgery for rectal neoplastic lesions

**DOI:** 10.1007/s00464-026-12694-9

**Published:** 2026-03-02

**Authors:** Alberto Arezzo, Giovanni Distefano, Carlo A. Ammirati, Michele Barbiero, Mario Morino

**Affiliations:** Department of Surgical Sciences, University of Turin, Corso Bramante 88, 10126 Turin, Italy

**Keywords:** Endorectal ultrasound, TEM, Rectal cancer, Local excision, Diagnostic accuracy, Learning curve

## Abstract

**Introduction:**

Endorectal ultrasound (EUS) is an essential tool for local staging of rectal neoplasia; however, its diagnostic accuracy in distinguishing non-invasive from invasive lesions before transanal endoscopic microsurgery (TEM) remains a matter of debate.

**Methods:**

A retrospective analysis of 1,000 consecutive EUS examinations performed before TEM between 1993 and 2025 was conducted using a prospectively maintained database. EUS levels (0–3) were correlated with the final histopathological outcome. Lesions were categorised as non-invasive (LGD, HGD, Tis) or invasive (pT1–pT3). Diagnostic metrics—sensitivity, specificity, positive predictive value (PPV), negative predictive value (NPV), and accuracy—were calculated overall and across three chronological periods. Separate analyses were performed for post-neoadjuvant (ypT03) and post-endoscopic resection groups.

**Results:**

Among 883 evaluable EUS studies for the primary analysis (non-invasive vs invasive pT1–pT3), overall sensitivity was 86.4%, specificity 64.9%, PPV 81.5%, NPV 72.7%, and accuracy 78.7%. All indices improved over time, with accuracy rising from 62.9% in early cases to 73.9% in the most recent period. In the post-neoadjuvant group (*n* = 47), sensitivity remained high (89.7%), but specificity was low (33.3%), likely due to overstaging related to fibrosis. In the post-endoscopic resection group (*n* = 70), the apparent accuracy was 44.3%, suggesting a high rate of false-positive invasion predictions.

**Conclusions:**

EUS before TEM shows good overall accuracy and excellent reliability for excluding deep invasion, with progressive improvement over the past 3 decades. While overstaging remains a limitation in post-treatment and non-dysplastic lesions, EUS continues to play a pivotal role in selecting candidates for organ-preserving rectal surgery. In post-endoscopic resection scars and post-neoadjuvant rectum, EUS findings should be interpreted cautiously and integrated with MRI/endoscopic morphology.

Endorectal ultrasound (EUS) is a crucial tool in the preoperative assessment of rectal neoplastic lesions, providing high-resolution imaging of the rectal wall layers and surrounding structures. Since its introduction in the late 1980s, EUS has been widely used to assess tumour invasion depth and the presence of perirectal nodal disease, guiding decisions between local excision and radical resection [1]. Its capacity to differentiate between mucosal and submucosal infiltration makes it particularly valuable for identifying early lesions suitable for organ-preserving approaches such as transanal endoscopic microsurgery (TEM), transanal minimally invasive surgery (TAMIS), and other local excision techniques.

While benign adenomas or carcinoma in situ can be safely managed with local excision [2], invasive adenocarcinomas—particularly those penetrating beyond the superficial submucosa—require radical resection to guarantee oncological safety [3]. In this delicate balance between oncological thoroughness and functional preservation, EUS has traditionally been regarded as the primary diagnostic tool.

Although widely used, the diagnostic reliability of EUS remains a topic of ongoing debate. Reported accuracy varies significantly across studies [4, 5], mainly due to differences in operator experience, probe technology, patient selection, and histopathological criteria. Overstaging benign or purely mucosal lesions is common and can lead to overtreatment, while understaging early submucosal invasion may compromise oncological safety. Variability among observers, tumour morphology, and the impact of neoadjuvant therapy also contribute to inconsistent clinical performance.

Furthermore, EUS technology has undergone significant evolution over the past thirty years. Early assessments employed rigid transrectal echoendoscopes with limited frequency and spatial resolution. Subsequent developments introduced flexible high-frequency probes and colour Doppler capabilities, enhancing image quality and lesion characterisation [6]. Alongside these technological advances, standardisation of staging criteria and increased operator training have gradually improved diagnostic reproducibility.

Most existing studies, however, are based on small or diverse cohorts, often limited to specific tumour stages or treatment settings. Few large series have systematically compared EUS findings with final histopathological results across the entire spectrum of rectal neoplasia, including benign dysplastic adenomas, carcinoma in situ, invasive adenocarcinoma, and post-neoadjuvant (ypT) stages [7]. Furthermore, little is known about how diagnostic performance has evolved over time, reflecting both technological advancements and the increasing expertise in high-volume referral centres.

This study aims to assess the diagnostic accuracy of 1,000 endorectal ultrasound examinations performed before transanal endoscopic microsurgery (TEM) for rectal neoplastic lesions. It provides a detailed comparison with final histopathology and explores how EUS performance has improved across three decades of clinical practice. By analysing an extensive, prospectively maintained database, the study aims to clarify the true value and limitations of EUS across various clinical scenarios, including early adenomas, invasive cancers, post-neoadjuvant lesions, and post-endoscopic resection cases.

## Materials and methods

### Study design and population

A retrospective observational study was conducted using a prospectively maintained database of patients who underwent transanal endoscopic microsurgery (TEM) for rectal neoplastic lesions at a single tertiary referral centre between 1993 and 2025. All cases with available preoperative endorectal ultrasound (EUS) examination and corresponding final histopathological diagnosis were included. Patients with incomplete or technically inadequate EUS data were excluded from the analysis.

A total of 1,000 consecutive EUS examinations were analysed. Demographic and clinical data, operative reports, and definitive histology were obtained from institutional electronic records. The study adhered to the STROBE guidelines for observational studies and received institutional review board approval [8].

Throughout the study period, patients were evaluated within an organ-preservation pathway combining endoscopy with biopsy and imaging. Pelvic MRI has become increasingly integrated into routine assessment in recent years, in line with evolving guidelines and local practice, and is interpreted in conjunction with EUS within a multidisciplinary setting to optimise staging and treatment selection. Importantly, the present study evaluates the diagnostic performance of EUS within this TEM-based pathway, while the reference standard for all accuracy analyses remains the final histopathology of the TEM specimen.

### Clinical pathway and indication for TEM

In our institution, patients referred for organ-preserving management of rectal neoplastic lesions undergo a standardised preoperative assessment, including endoscopy with biopsy and imaging (EUS and pelvic MRI, when available, according to the era and local practice). Treatment allocation (local excision vs radical resection) is determined within a colorectal multidisciplinary setting and is individualised based on endoscopic morphology, histology, imaging findings, lesion level/extent, patient comorbidities, and patient preference. TEM is offered as definitive treatment for lesions deemed suitable for local excision (suspected non-invasive neoplasia and selected early cancers), and it is also used for full-thickness excision to achieve definitive histopathological staging in selected cases with uncertain or discordant preoperative assessment (excision-as-staging). Accordingly, the present cohort includes patients with EUS categories suggestive of invasion (EUS 1–3) who underwent TEM because of discordant benign/superficial histology, anticipated overstaging, high operative risk or refusal of radical surgery, and/or a planned strategy of completion radical surgery if high-risk features (e.g., ≥ pT2, adverse histology, positive margins) were confirmed on the TEM specimen.

During the study period, TEM represented the standard platform for full-thickness local excision in our unit. Other approaches (e.g., TAMIS and endoscopic resections such as EMR/ESD) have been used in selected cases according to availability and indications; however, they were not included in this analysis to ensure a homogeneous reference standard based on full-thickness TEM specimens. Lesions assessed after prior endoscopic resection were analysed separately (post-endoscopic resection group).

### Endorectal ultrasound procedure

All EUS examinations were conducted using either rigid or flexible probes (initially 7.5 MHz, then upgraded to 12–15 MHz) following standard protocols. Patients were examined in the left lateral decubitus position, and the rectal wall was scanned in four quadrants to ensure complete visualisation of the lesion and surrounding layers.

EUS findings were categorised into four groups based on the extent of wall infiltration.

- EUS 0: lesion confined to the mucosa (no submucosal invasion)
- EUS 1: limited submucosal invasion
- EUS 2: deep submucosal or muscularis propria involvement
- EUS 3: transmural or extramural invasion

Endorectal ultrasound examinations were performed using rigid or flexible probes, according to standardised protocols, by operators with dedicated expertise in rectal endosonography. Given the long study period, examinations were performed by experienced gastroenterologists at our institution or, in selected earlier cases, by gastroenterologists at referring centres before patient referral; in the later and larger part of the series, EUS was performed predominantly with flexible instruments by expert gastroenterologists at our hospital. Formal interobserver variability was not assessed. In the early years of the series, rigid probes were mainly used, while high-resolution flexible probes became standard after 2010.

### Histopathological classification

The final diagnosis was derived from full-thickness TEM specimens, evaluated by specialised gastrointestinal pathologists in accordance with the WHO and Vienna classifications [9, 10]. Histopathological results were categorised as follows:

- Non-invasive lesions: low-grade dysplasia (LGD), high-grade dysplasia (HGD), and carcinoma in situ (Vienna 4.2–4.4, pTis)
- Invasive carcinomas: pT1 (submucosal invasion stratified as sm1, sm2, sm3), pT2, and pT3
- Post-neoadjuvant therapy group: ypT0, ypT1, ypT2, ypT3
- Post-endoscopic resection group

For the primary analysis, non-invasive lesions were compared with invasive carcinomas (pT1–pT3), while ypT and post-endoscopic resection cases were analysed separately. To address the clinical decision-making relevant to organ-preserving strategies, we performed a secondary analysis using a “beyond local excision” endpoint. Two clinically meaningful dichotomies were considered: (i) advanced disease defined as ≥ pT2 versus ≤ pT1 (where pT1 is managed within an organ-preservation/local excision pathway in selected patients); and (ii) a risk-proxy definition of beyond local excision as ≥ pT1sm2 (pT1sm2–sm3, pT2–pT3) versus ≤ pT1sm1 (non-invasive lesions plus pT1sm1).

### Diagnostic definitions

EUS results were considered positive for invasion when categorised as EUS 1–3, and negative (non-invasive) when EUS 0. For the clinically oriented analyses, EUS classifications were evaluated against the above dichotomised histopathological endpoints; diagnostic indices (sensitivity, specificity, PPV, NPV, accuracy) were computed as for the primary analysis.

For each case, diagnostic performance was evaluated by cross-matching EUS results with definitive histopathology. True positive (TP), true negative (TN), false positive (FP), and false negative (FN) values were computed accordingly.

Diagnostic accuracy measures included:

$$Sensitivity \, = \, TP/\left( {TP + FN} \right)$$

$$Specificity \, = \, TN/\left( {TN + FP} \right)$$

$$Positive \, Predictive \, Value \, \left( {PPV} \right) \, = \, TP/\left( {TP \, + \, FP} \right)$$

$$Negative \, Predictive \, Value \, \left( {NPV} \right) \, = \, TN/\left( {TN + FN} \right)$$

$$Accuracy \, = \, \left( {TP + TN} \right)/\left( {TP + TN + FP + FN} \right)$$

where

$$TP \, = \, True \, Positive, \, TN \, = \, True \, Negative, \, FP \, = \, False \, Positive, \, FN \, = \, False \, Negative$$

In other words:

Sensitivity is the ability of the test to identify patients with the disease correctly.

Specificity is the ability of the test to identify those without the disease correctly.

PPV (Positive Predictive Value) is the probability that a patient with a positive test truly has the disease.

NPV (Negative Predictive Value) is the probability that a patient with a negative test truly does not have the disease.

Accuracy is the overall proportion of correctly classified cases.

### Statistical analysis

Continuous variables were expressed as median (interquartile range, IQR), and categorical variables as counts and percentages.

When comparing the predictive role of EUS in distinguishing non-invasive from invasive neoplasms, diagnostic performance was evaluated for the overall cohort and for three chronological subgroups of approximately 295 cases each, ordered by date of surgery, to assess the temporal development of EUS accuracy. In addition, diagnostic performance was recalculated for the clinically oriented endpoints (≥ pT2 vs ≤ pT1; and ≥ pT1sm2 vs ≤ pT1sm1).

Separate analyses were conducted for post-neoadjuvant (ypT) and post-endoscopic resection cases.

Comparisons between groups were conducted using the *χ*^2^ or Fisher’s exact test, as appropriate. Statistical significance was defined as *p* < 0.05. Predictive values and prevalence. Positive and negative predictive values (PPV/NPV) were reported because they are clinically informative within the TEM-based organ-preservation pathway represented by this cohort; however, PPV and NPV are prevalence-dependent and should not be extrapolated to unselected populations. To provide prevalence-independent measures of test performance, we additionally calculated likelihood ratios (LR + and LR −) and the diagnostic odds ratio (DOR).

Confidence intervals. 95% confidence intervals (CI) were computed for sensitivity, specificity and overall accuracy using the Wilson method.

All analyses were carried out using IBM SPSS Statistics version 29.0 and Python 3.12 for data visualisation [11].

## Results

### Study population

A total of 1,000 consecutive patients who underwent preoperative endorectal ultrasound (EUS) before transanal endoscopic microsurgery (TEM) were included. This TEM-based cohort reflects our organ-preservation pathway, in which TEM is used both as definitive local excision and, in selected cases with uncertain/discordant staging, as full-thickness excision for definitive histopathological staging; therefore, patients with EUS categories suggestive of invasion (EUS 1–3) are also represented.

Of 1,000 consecutive TEM cases with EUS and final histology, 883 were included in the primary analysis (non-invasive vs invasive pT1–pT3). The post-neoadjuvant cases (ypT0–3, *n* = 47) and post-endoscopic resection scar/no-dysplasia cases (*n* = 70) were analyzed separately due to altered wall anatomy/fibrosis and different clinical meaning of EUS findings.

The median age was 67 years (range 33–92), with a slight male predominance (58%).

Final histopathology revealed a full spectrum of rectal neoplastic lesions, from adenomas with dysplasia to invasive adenocarcinomas.

The cross-tabulation of EUS and histopathological diagnosis is presented in Table 1.

**Table 1:** Correlation between endorectal ultrasound (EUS) and final histopathology

EUS	HGD	LGD	NO DYSPLASIA	Tis 4.2	Tis 4.4	pT1sm1	pT1sm2	pT1sm3	pT2	pT3	ypT0	ypT1	ypT2	ypT3	Total
0	279	111	33	10	13	8	9	9	21	8	4	2	2	0	509
1	120	46	37	15	13	31	25	20	36	5	2	4	2	0	356
2	14	8	0	2	2	6	5	12	30	11	2	4	15	4	115
3	2	1	0	1	0	0	2	0	4	4	0	0	5	1	20
Total	415	166	70	28	28	45	41	41	91	28	8	10	24	5	1000

Most non-invasive lesions (LGD, HGD, Tis) are classified within the EUS 0–1 categories, while advanced carcinomas (pT2–pT3) are mainly found in the EUS 2–3 categories.

### Overall diagnostic performance

When lesions were grouped as non-invasive (LGD, HGD, Tis) versus invasive (pT1–pT3), endorectal ultrasound demonstrated a sensitivity of 86.4% and a specificity of 64.9% (Table 2). The positive predictive value (PPV) was 81.5%, while the negative predictive value (NPV) reached 72.7%. The overall diagnostic accuracy for distinguishing non-invasive from invasive disease was 78.7%.

**Table 2:** Diagnostic performance of endorectal ultrasound (EUS) for distinguishing non-invasive from invasive rectal lesions (*n* = 883)

	Histology: non-invasive	Histology: invasive (pT1–pT3)	Total
EUS 0 (non-invasive)	TN = 205	FN = 77	282
EUS 1–3 (invasive)	FP = 111	TP = 490	601
Total	316	567	883

Metric	Value (%)
Sensitivity	86.4
Specificity	64.9
Positive predictive value (PPV)	81.5
Negative predictive value (NPV)	72.7
Overall accuracy	78.7
LR +	2.46
LR-	0.21
DOR	11.75

*TP* True positive, *FP* False positive

Diagnostic performance of endorectal ultrasound (EUS) for distinguishing non-invasive from invasive rectal lesions (*n* = 883)

Taken together, these results indicate that EUS was particularly reliable in ruling out deep invasion, whereas overstaging of superficial lesions accounted for most false-positive findings.

When a clinically oriented endpoint was adopted—considering ≥ pT2 as “beyond local excision” and grouping ≤ pT1 within the organ-preservation/local excision pathway—EUS (EUS 0 vs EUS 1–3) showed sensitivity 75.6%, specificity 57.5%, PPV 21.7%, NPV 93.8%, and accuracy 59.9% (Table 3). This indicates that an EUS 0 result reliably excludes ≥ pT2 disease, whereas EUS frequently overcalls superficial invasion, explaining the low PPV.

**Table 3:** Diagnostic performance of endorectal ultrasound (EUS) for distinguishing non-invasive from invasive rectal lesions (*n* = 883)

	Histology: ≤ pT1	Histology: ≥ pT2	Total
EUS 0 (non-invasive)	TN = 439	FN = 29	468
EUS 1–3 (invasive)	FP = 325	TP = 90	415
Total	764	119	883

Metric	Value (%)
Sensitivity	75.6
Specificity	57.5
Positive predictive value (PPV)	21.7
Negative predictive value (NPV)	93.8
Overall accuracy	59.9
LR +	1.78
LR-	0.42
DOR	4.19

*TP* True positive, *FP* False positive

Diagnostic performance of endorectal ultrasound (EUS) for distinguishing non-invasive from invasive rectal lesions (*n* = 883)

Using a risk-proxy definition of beyond local excision (≥ pT1sm2 vs ≤ pT1sm1), performance was sensitivity 76.6%, specificity 61.7%, PPV 37.1%, NPV 89.9%, and accuracy 65.1% (Table 4), supporting the clinical value of EUS as a triage tool in an organ-preservation pathway.

**Table 4:** Diagnostic performance of endorectal ultrasound (EUS) for distinguishing non-invasive from invasive rectal lesions (*n* = 883)

	Histology: ≤ pT1sm1	Histology: ≥ pT1sm2	Total
EUS 0 (non-invasive)	TN = 421	FN = 47	468
EUS 1–3 (invasive)	FP = 261	TP = 154	415
Total	682	201	883

Metric	Value (%)
Sensitivity	76.6
Specificity	61.7
Positive predictive value (PPV)	37.1
Negative predictive value (NPV)	89.9
Overall accuracy	65.1
LR +	2.00
LR-	0.38
DOR	5.29

*TP* True positive, *FP* False positive

Diagnostic performance of endorectal ultrasound (EUS) for distinguishing non-invasive from invasive rectal lesions (*n* = 883)

The detailed 2 × 2 contingency table is shown in Table 2.

### Temporal evolution

**Fig. 1:**
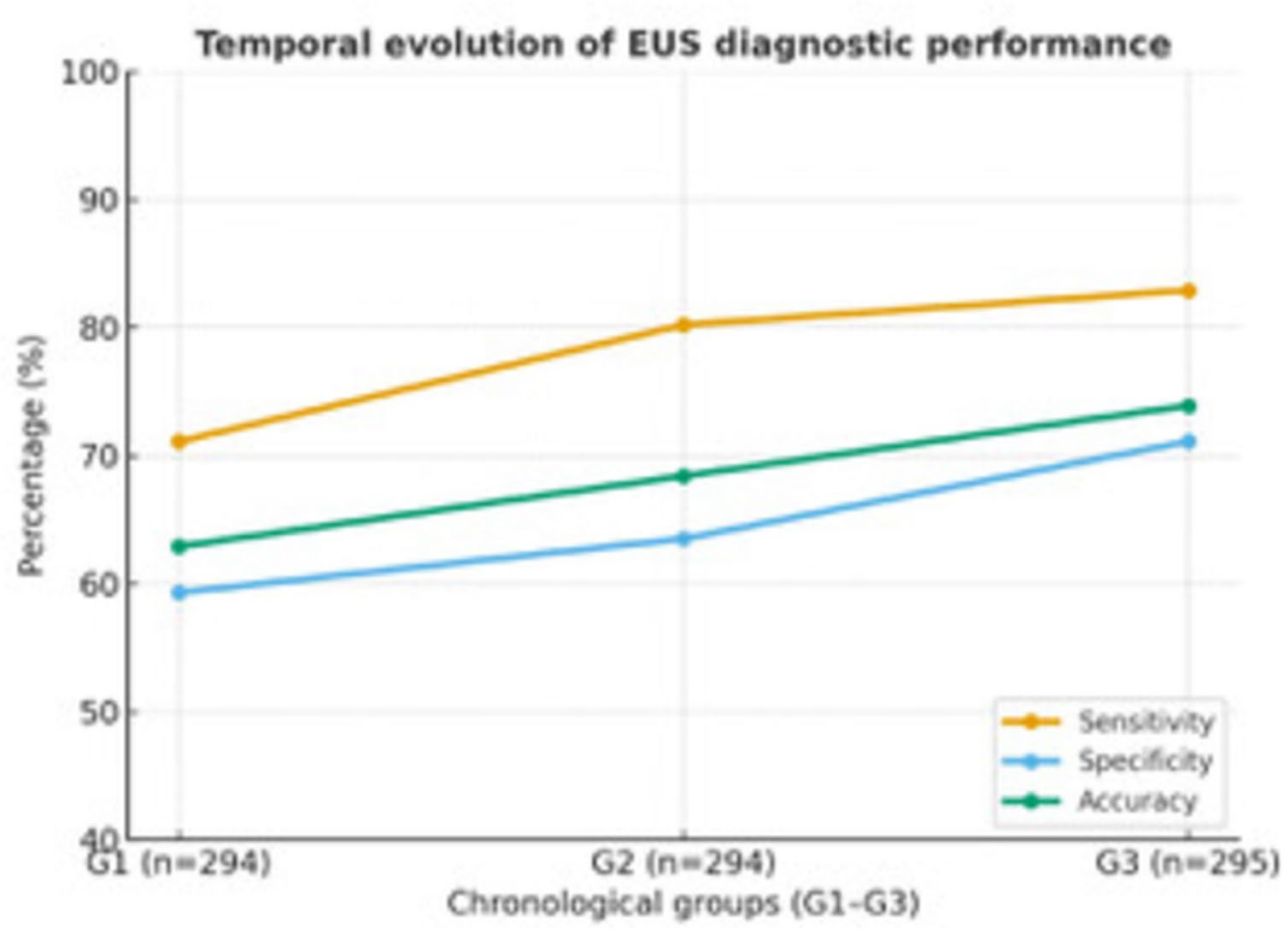
Temporal evolution of the diagnostic performance of endorectal ultrasound (EUS) in distinguishing non-invasive from invasive rectal lesions. Patients were divided into three chronological groups (G1–G3), each comprising approximately 295 cases. All diagnostic indices improved progressively over time, with sensitivity rising from 71 to 83%, specificity from 59 to 71%, and overall accuracy from 63 to 74%

Patients were separated into three chronological groups, each comprising approximately 295 cases, representing the early (group 1), intermediate (group 2), and most recent (group 3) phases of the 30-year experience. This division allowed for the assessment of the progressive development of EUS performance over time (Table 5, Fig. 1).

**Table 5:** Temporal evolution of diagnostic performance of endorectal ultrasound (EUS) across three chronological periods

Time period	Year ranges	Sensitivity	Specificity	PPV	NPV	Accuracy
G1 (n 294)	1993–2008	71.1%	59.3%	43.5%	82.3%	62.9%
G2 (n 294)	2008–2016	80.2%	63.5%	47.6%	88.6%	68.4%
G3 (n 295)	2016–2025	82.9%	71.1%	47.2%	93.0%	73.9%

All diagnostic indices gradually improved over the three decades, with the most notable rise seen in specificity and overall accuracy.

Over time, EUS sensitivity for detecting invasive lesions increased from 71 to 83%, while specificity rose from 59 to 71%. The overall accuracy improved from 63% in the early years to 74% in the most recent period, confirming an apparent learning curve driven by technological advances and operator experience.

### Post-neoadjuvant group (ypT0–3)

Among patients receiving neoadjuvant therapy, 47 cases exhibited residual disease or complete pathological response (ypT0–3).

When ypT0 was regarded as non-invasive and ypT1–3 as invasive, EUS showed high sensitivity (89.7%) and positive predictive value (94.6%), with an overall accuracy of 85.7%. In contrast, specificity (33.3%) and negative predictive value (20.0%) remained limited due to fibrosis-related overstaging (Table 6).

**Table 6:** Diagnostic performance of endorectal ultrasound (EUS) in post-neoadjuvant (ypT0 3) rectal cancer cases (*n* = 47)

Metric	Value (%)
Sensitivity	89.7
Specificity	33.3
PPV	94.6
NPV	20.0
Accuracy	85.7

ypT0 lesions were considered non-invasive, whereas ypT1–ypT3 were classified as invasive

EUS maintained high sensitivity and positive predictive value (PPV), although specificity and negative predictive value (NPV) remained limited due to overstaging related to fibrosis

The analysis of the two chronological halves (P1 vs. P2, with 23 vs 24 cases) showed progressive improvement: specificity increased from 0 to 50%, and accuracy improved from 81% to 90.5%.

These findings indicate that, although EUS maintains high sensitivity after neoadjuvant treatment, fibrotic changes might cause overstaging and a low NPV.

### Post-endoscopic resection group

In lesions classified as “no dysplasia” in the final pathological report after post-endoscopic resection, EUS often overestimated invasion.

The distribution of EUS categories was 44.3% for EUS 0 (31/70), 31.4% for EUS 1 (22/70), 14.3% for EUS 2 (10/70), and 10.0% for EUS 3 (7/70), indicating a predominant tendency to overstaging non-dysplastic lesions after endoscopic resection (Table 7).

**Table 7:** Distribution of endorectal ultrasound (EUS) findings in lesions classified as “no dysplasia” after post-endoscopic resection (*n* = 70)

EUS	N	%
0	31	44.3
1	22	31.4
2	10	14.3
3	7	10.0

Accordingly, the apparent accuracy was 44.3%, while 55.7% were false-positive predictions of invasion.

These findings confirm that EUS often overestimates benign or post-procedural mucosal irregularities, especially in scars after endoscopic resection or when inflammatory changes are present.

Across all subgroups, EUS showed:

- Consistently high sensitivity (≈73–90%) and NPV (80–90%),
- Specificity improves with time,
- Overall accuracy rises from 63 to 74%.

These data collectively emphasise that EUS remains a reliable tool for excluding deep invasion before TEM, and that technological and experiential advances have greatly improved its ability to differentiate over time.

## Discussion

This study, which includes 1,000 consecutive patients who underwent endorectal ultrasound (EUS) before transanal endoscopic microsurgery (TEM), is among the most comprehensive single-centre analyses assessing the diagnostic performance of EUS across the full spectrum of rectal neoplastic lesions. The findings confirm that EUS provides good overall accuracy, with notably high sensitivity and negative predictive value (NPV), and also demonstrate a clear trend of improvement over time.

### Diagnostic value of EUS before TEM

EUS remains the most commonly used tool for assessing rectal wall invasion depth in early rectal cancer [12], despite ongoing advances in other preoperative imaging methods [13–15]. In our series, EUS differentiated between invasive and non-invasive lesions with a sensitivity of 86.4% and a specificity of 64.9% for the primary endpoint (Table 2). These findings are consistent with previous meta-analyses, which report accuracy rates ranging from 65 to 80% for T-staging of rectal cancer [16]. From a clinical standpoint, the most actionable question is whether EUS can exclude disease beyond local excision. In our cohort, an EUS 0 finding provided a high NPV for ≥ pT2 disease (93.8%), supporting its role as a triage tool in organ-preservation pathways, while acknowledging a tendency to overstage superficial lesions.

Clinically, the strength of EUS in a TEM-based organ-preservation pathway mainly lies in its ability to rule out disease beyond local excision: an EUS 0 result showed a high NPV for ≥ pT2 disease (93.8%; Table 3) and for ≥ pT1sm2 (89.9%; Table 4), supporting its role as a triage tool, while acknowledging a tendency to overstage superficial lesions. However, false-positive results—overstaging mucosal or superficial lesions as invasive—remain a limitation, potentially leading to overtreatment or unnecessary delays in TEM.

### Evolution over three decades

A key finding of this study is the continuous improvement in diagnostic performance over time. Across three chronological periods of approximately 295 cases each, all diagnostic measures—sensitivity, specificity, PPV, NPV, and accuracy—showed consistent improvement, with accuracy rising from 63 to 74%. PPV and NPV should be interpreted in the context of a TEM-selected organ-preservation pathway, in which case mix and disease prevalence differ from those in unselected rectal neoplasia cohorts. Accordingly, we also report prevalence-independent measures (LR + and LR −), which may better support transferability across settings with different pre-test probabilities. In this framework, the high NPV observed for “beyond local excision” endpoints supports the role of EUS as a rule-out/triage tool, while the modest LR + reflects a tendency to overstage superficial disease.

This trend likely reflects several factors: (i) the evolution of probe technology from early rigid to high-frequency flexible transducers; (ii) refined techniques with better spatial orientation and image interpretation; and (iii) the accumulation of operator experience in a high-volume referral setting.

These findings support the idea of a learning curve for EUS in rectal staging [17], similar to that described for transanal surgery itself and for the broader evolution of minimally invasive colorectal techniques [18, 19]. Once initial proficiency is achieved, EUS can reliably and consistently provide accurate information to guide TEM case selection.

### Clinical implications in local excision strategies

Achieving precise preoperative staging is vital in deciding whether to choose local excision or radical resection. According to international guidelines [20–22], TEM or other transanal techniques are advised for lesions limited to the mucosa or with minimal submucosal invasion (T1 sm1) and with favourable histological characteristics, such as G1–G2 grading, submucosal invasion sm2-sm3, lymph-vascular invasion. Understaging these lesions could jeopardise oncological safety, whereas overstaging might lead to unnecessary radical surgery.

The current data show that EUS is a reliable safeguard against undertreatment because of its high sensitivity and negative predictive value (NPV). When EUS classifies a lesion as non-invasive (EUS 0), the chance of actual superficial disease exceeds 85%. However, a positive EUS result (EUS ≥ 1) does not automatically indicate deep invasion; in this case, combining EUS with endoscopic morphology, MRI, and biopsies obtained during endoscopic resection may enhance patient selection.

In contemporary practice, staging is increasingly based on a combined interpretation of MRI and EUS, rather than either modality alone. Our results should therefore be interpreted as the performance of EUS within a real-world TEM-based organ-preservation pathway, in which MRI provides complementary information, particularly for deeper invasion and features relevant to radical treatment planning.

### Performance in the post-neoadjuvant setting

Among the subgroup of 47 patients who underwent EUS after neoadjuvant therapy, the technique maintained high sensitivity (89.7%) and PPV (94.6%), confirming its effectiveness in detecting residual invasive disease. However, specificity was only 33% and NPV was 20%, indicating frequent overstaging due to fibrosis and inflammation.

These findings are consistent with previous reports indicating that post-treatment tissue remodelling significantly decreases EUS accuracy [23]. In our temporal analysis, the most recent cases in the second period demonstrated improved discrimination, with specificity increasing to 50% and overall accuracy approaching 91%. This improvement reflects a more cautious interpretation of hypoechoic fibrosis, improved correlation with MRI, and increased operator awareness of post-therapeutic artefacts.

Therefore, while EUS may not reliably differentiate between complete and near-complete responses after chemoradiation, it remains useful in confirming persistent infiltration, particularly when used in conjunction with other modalities.

### The post-endoscopic resection dilemma

Among the 70 cases ultimately classified as post-endoscopic resection, EUS overestimated the depth of invasion in more than half (56%). This overstaging is clinically significant because benign mucosal irregularities, post-polypectomy scars, or chronic inflammation can resemble submucosal thickening.

Our data confirm that EUS has limited specificity in such contexts: only 44% of these cases were correctly classified as non-invasive (EUS 0). Recognising this limitation is essential to prevent the overtreatment of endoscopically benign lesions. In these scenarios, a strategy based on initial endoscopic resection or surveillance, supported by histological confirmation, may be preferable to EUS-based surgical decisions [2].

### Comparison with previous literature

Previous studies have reported highly variable accuracies for EUS in rectal staging, depending on tumour stage and lesion characteristics. Early works by Morson and Hermanek established histopathological correlations that remain valid benchmarks for assessing local invasion [24, 25].

Subsequent series—such as those by Yamaguchi et al. (2004) and Lee et al. (2019)—reported sensitivities ranging from 70 to 90% [26–28], consistent with our findings. However, most of these studies involved fewer than 200 patients and did not investigate temporal changes.

Our series is unique in combining a large sample size, a comprehensive histological spectrum, and a longitudinal analysis spanning over 30 years. The documented improvement in EUS performance over time emphasises the importance of experience, standardisation, and ongoing quality monitoring.

### Limitations

The main limitations of this study are its retrospective design and potential selection bias, as only patients referred for TEM were included in the analysis. As this is a TEM-selected population from an organ-preservation pathway, the case mix differs from that of unselected rectal neoplasia. The operator-dependent nature of EUS, equipment variability, and changes in reporting over three decades introduce heterogeneity that cannot be entirely corrected.

Notably, EUS technology itself has undergone significant evolution over the past 30 years. Early examinations were often performed using transrectal rigid echoendoscopy (TRUS), whereas in later years, high-resolution flexible endoscopic ultrasound probes became the standard practice. Additionally, not all EUS examinations were performed at the same institution; many patients were referred to our centre after staging at their referring hospitals, and in most cases, EUS was not repeated prior to TEM. These differences in device type, examiner expertise, and institutional protocols naturally contributed to variability in image quality and interpretation.

Interobserver variability and the lack of centralised image review may have affected the classification of earlier cases. However, the consistency of the observed time-related improvement and the strength of the prospective database reinforce the validity and broader applicability of the findings.

Finally, a limitation is the lack of systematic MRI data across the entire 30-year period, precluding a formal comparative or combined-modality accuracy analysis; however, the reference standard was uniform (TEM histopathology), and the study reflects how EUS was applied within the evolving clinical pathway.

### Clinical and research perspectives

From a clinical perspective, these results endorse the ongoing use of EUS as a primary tool for evaluating rectal neoplasia before TEM, especially for excluding deep invasion.

Future efforts should focus on integrating EUS findings into multimodal predictive models, combining MRI, digital pathology, and artificial intelligence–assisted interpretation to improve accuracy and reproducibility.

In research, extensive prospective registries with standardised EUS criteria and digital image archiving could help validate these findings and establish thresholds for clinical decision-making in organ-preserving rectal surgery.

Over three decades and 1,000 preoperative examinations, endorectal ultrasound (EUS) has demonstrated good overall diagnostic accuracy and an excellent negative predictive value in assessing rectal neoplastic lesions before transanal endoscopic microsurgery (TEM). Continuous technological refinement and growing operator expertise have progressively improved its performance, confirming EUS as a key step in selecting patients suitable for safe, organ-preserving local excision.

However, the diagnostic value of EUS is limited in post-radiotherapy and post-endoscopic resection settings, where fibrosis, scarring, and mucosal distortion frequently mimic tumour invasion and lead to overstaging. In these contexts, clinical decision-making should rely on a multimodal approach that integrates magnetic resonance imaging, endoscopic evaluation, and histopathological findings rather than on EUS alone.
