# Author Correction: Diagnostic accuracy of 1,000 endorectal ultrasounds before transanal endoscopic microsurgery for rectal neoplastic lesions

**DOI:** 10.1007/s00464-026-12928-w

**Published:** 2026-06-01

**Authors:** Alberto Arezzo, Giovanni Distefano, Carlo A. Ammirati, Michele Barbiero, Mario Morino

**Affiliations:** Department of Surgical Sciences, University of Turin, Corso Bramante 88, 10126 Turin, Italy

**Author Correction to: Surgical Endoscopy (2026) 40:4004–4013** 10.1007/s00464-026-12694-9

In the Methods section of the original article, the sentence referring to the operators performing or reviewing endorectal ultrasound examinations may have inaccurately suggested that the entire series was performed or reviewed exclusively by colorectal surgeons trained in endosonography.

The original sentence:

All EUS studies were conducted or reviewed by experienced colorectal surgeons trained in endosonography, predominantly by two main operators across the study period.

should be corrected as follows:

Endorectal ultrasound examinations were performed using rigid or flexible probes, according to standardized protocols, by operators with dedicated expertise in rectal endosonography. Given the long study period, examinations were performed by experienced gastroenterologists at our institution or, in selected earlier cases, by gastroenterologists at referring centres before patient referral; in the later and larger part of the series, EUS was performed predominantly with flexible instruments by expert gastroenterologists at our hospital.

This correction clarifies the professional background of the operators and emphasizes that the key determinant of examination quality was specific expertise in rectal endosonography rather than specialty background. The correction does not affect the data, analyses, results, or conclusions of the article.

